# Comparison of axon extension: PTFE versus PLA formed by a 3D printer

**DOI:** 10.1515/biol-2022-0031

**Published:** 2022-03-31

**Authors:** Naofumi Kawai, Mizuki Bando, Kento Yuasa, Masayuki Shibasaki

**Affiliations:** Department of Anesthesiology, Kyoto Prefectural University of Medicine, 465 Kajiicho, Kamigyo-Ku, Kyoto-Shi, Kyoto-Fu 604-8404, Japan; Department of Anesthesiology, Akashi City Hospital, 1-33, Takasho-Machi, Akashi-Shi, Hyogo-Ken, 673-8501, Japan

**Keywords:** 3D printer, PLA, PTFE, Campenot chamber, cell culture

## Abstract

Three-dimensional (3D) printers mainly create 3D objects by stacking thin layers of material. The effect of the tools created using the fused deposition modeling (FDM) 3D printer on nerve cells remains unclear. In this study, the effects of polytetrafluoroethylene (PTFE) models and two different types of polylactic acid (PLA) models (white or natural), were created using the FDM 3D printer on axon extension were compared using the Campenot chamber. Neurons were isolated from the dorsal root ganglia and added to the central compartment of the Campenot chambers after isolation, processing, and culturing. On day 7, after the initiation of the culture, the difference of the axon extensions to the side compartments of each group was confirmed. We also compared the pH and the amount of leakage when each of these chambers was used. The PLA was associated with a shorter axon extension than the PTFE (white *p* = 0.0078, natural *p* = 0.00391). No difference in the pH was observed (*p* = 0.347), but there was a significant difference on multiple group comparison (*p* = 0.0231) in the amount of leakage of the medium. PTFE was found to be a more suitable material for culturing attachments.

## 1 Introduction

In recent years, three-dimensional (3D) printer technology has attracted considerable attention. 3D printing is a technology that creates real objects based on a 3D model designed using computer-assisted design (CAD) software. In most cases, thin layers of material are piled up by hardening with ultraviolet (UV) radiation or melting with heat to create a substance based on the 3D model.

Generally, by standardizing tools, it is possible to perform experiments with high reproducibility; if the data of the 3D model can be shared, 3D printers enable us to experiment with high reproducibility.

The mainstream 3D printing method is fused deposition modeling (FDM) [1]. This involves stacking materials, such as polymer, molten at high temperatures as a thin layer, and the common materials used are polylactic acid (PLA) and acrylonitrile-butadiene-styrene (ABS).

Attempts were being made to develop devices for *in vitro* research in the field of cell culture, such as using a cell culture platform created by a 3D printer to culture fibroblasts [2]. There is also a strong interest in the safety of materials formed by 3D printing, and several studies have been conducted. Some studies conclude that it is harmful [3–8], but that some pretreatment may make it safe [5–7]. Some PLA devices for culturing neurons have been proposed [9]; however, their effects have not been sufficiently investigated.

Polytetrafluoroethylene (PTFE) is famous for its trade name, which is Teflon. It is useful due to its resistance to chemical substances, insulating properties, and the lowest coefficient of friction among solids. PTFE has high biosafety [10]. PLA is one of the most commonly used materials for biomedical applications [11]. It is derived from plants such as corn starch and is said to be biodegradable [12,13] and biosafe [14]. It is attracting attention as a carbon-neutral material with attractive properties; it is renewable, biocompatible, processable, and energy-saving [15] and used as medical implants and drug carriers [16,18]. It is approved by the US Food and Drug Administration for human use for sutures, bone implants, and screws, as well as formulations for sustained drug delivery and vaccine antigens (proteins, peptides, and DNA) [19]. On the other hand, when it is used as a biomaterial, its strong hydrophobic property can lead to an inflammatory response [20]. *In vitro*, while it was reported that Chinese hamster ovary cells showed good proliferation and adhesion to PLA [21], it has been reported that PLA suppresses fibroblast adhesion, elongation, and alkaline phosphatase activity in the early stage of culturing [22].

In this study, isolated neuron dividers developed by Dr. Campenot [23] (the so-called Campenot chambers) were created using PLA and an FDM 3D printer, and the effect of the divider on nerve cells was controlled by Campenot chambers made of PTFE, which is the commonly used material for Campenot chambers [23–25].

Then, after that experiment, the pH of the medium due to contact with each material with a pH monitor (LAQUAtwin pH-22B; HORIBA Advanced Techno, Kyoto, Japan) and the amount of leakage of each type of chamber were measured using an absorptiometer (SmartSpec3000; Bio-Rad Laboratories Inc, California, USA).

## 2 Materials and methods

### 2.1 3D printings

In this study, all PLA Campenot chambers were fabricated using the FDM 3D printer (Creator pro; Flashforge, Zhejiang, China).

**Figure 1:**
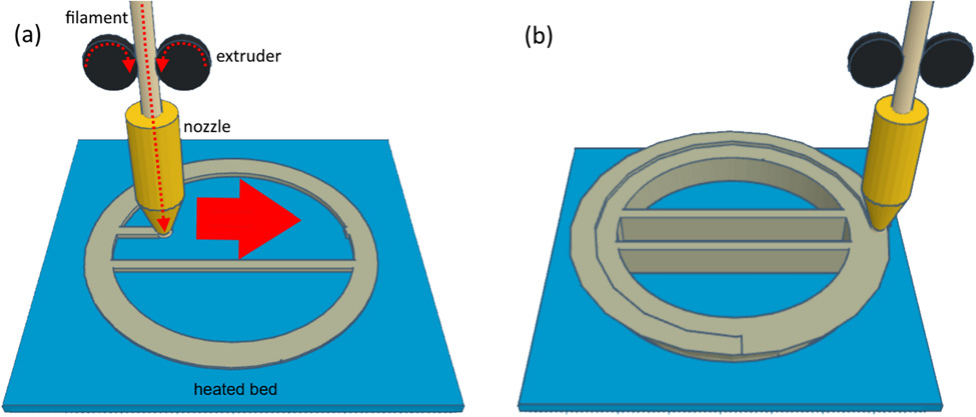
Three-dimensional printer principle. Filaments are made of polymer that is easy to melt by heat, such as PLA and ABS. They are loaded by an extruder with constant speed, and they pass through a heated nozzle. With the movement of the nozzle, a thin layer is drawn. The first layer is drawn on a heated bed (a). The heated bed stabilizes the forming model. Subsequent layers are drawn on the lower layer (b).

In an FDM 3D printer, a filament that is easily molten in the heat was passed through a nozzle that was heated to a constant temperature to create tools. The filament is fed at a constant speed using an extruder. This caused the heat-molten resin to constantly exit the tip of the nozzle, which drew a layer with thickness programmed on the heated bed (Figure 1a). The heated bed prevented the shrinkage of PLA and made it easier to attach the modeled object by warming the adhesive surface.

By stacking the layers, the desired shape was obtained (Figure 1b). Therefore, there must be a layer of scaffolding beneath the layer being drawn, which restricts the creation of tools with complex shapes. Therefore, when modeling an item with specific shape using CAD software, it is necessary to evaluate its formation. If not possible, it may be necessary to make plans, such as modeling support structures.

In this study, TinkerCAD (Autodesk, California, USA) was used to create the model and the stereolithography (STL) data. The STL data were translated using FlashPrint (Flashforge, Zhejiang, China) version 4.2.0 and saved with a filename extension of “x3” for 3D printing.

Two types of PLA filaments were used: white (Ender filament, Creality 3D, Shenzhen, China) and natural (PolyLite, Polymaker, Tokyo, Japan). Both filaments have a diameter of 1.75 mm. The conditions for the 3D printing are as follows: the temperature of the nozzle is 210°C and that of the heated bed is 60°C; the thickness of the first layer is 0.40 mm and that of the other layers is 0.20 mm; infill density of 100%; printing speed of 50 mm/s; the fan was run only during bottom printing; the nozzle diameter is 0.4 mm; and no auxiliary structures such as rafts, frames, or support materials were used.

In this experiment, PTFE and white PLA were first compared as white is the most basic color of PLA filaments, and then PTFE and natural PLA were compared in the next experiment; natural PLA has high purity [26].

The formed PLA chambers were washed with pure water three times, and before use, white PLA was autoclaved (121°C, 20 min) and natural PLA was UV sterilized (ultraviolet C [UVC], 15 W, 30 cm, 30 min).

### 2.2 Campenot chamber

**Figure 2:**
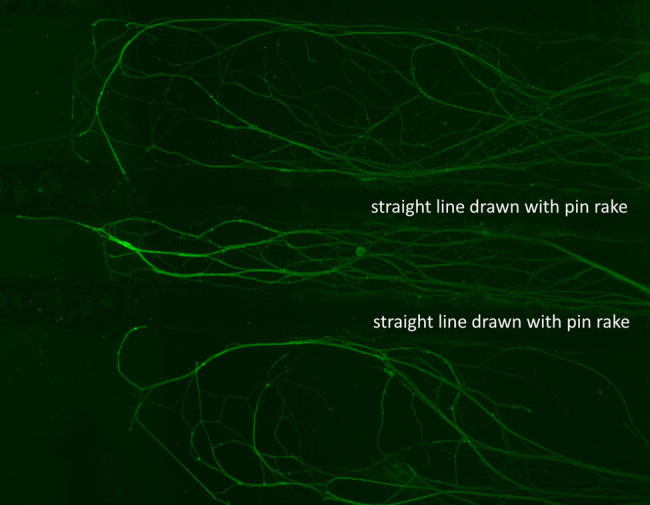
Straight lines drawn by pin rake prevent the unintended extension of the axon. The green structures in the picture are axons immunostained by anti-beta tubulin antibodies. This picture shows axons extending along the lines drawn with a pin rake.

A 6-well plate coated with poly-d-lysine/laminin was used for each culture. First, each well was scratched in parallel straight lines using a pin rake (Tyler Research, Alberta, Canada). These lines prevent the axon from extending in an unintended direction (Figure 2) [23].

**Figure 3:**
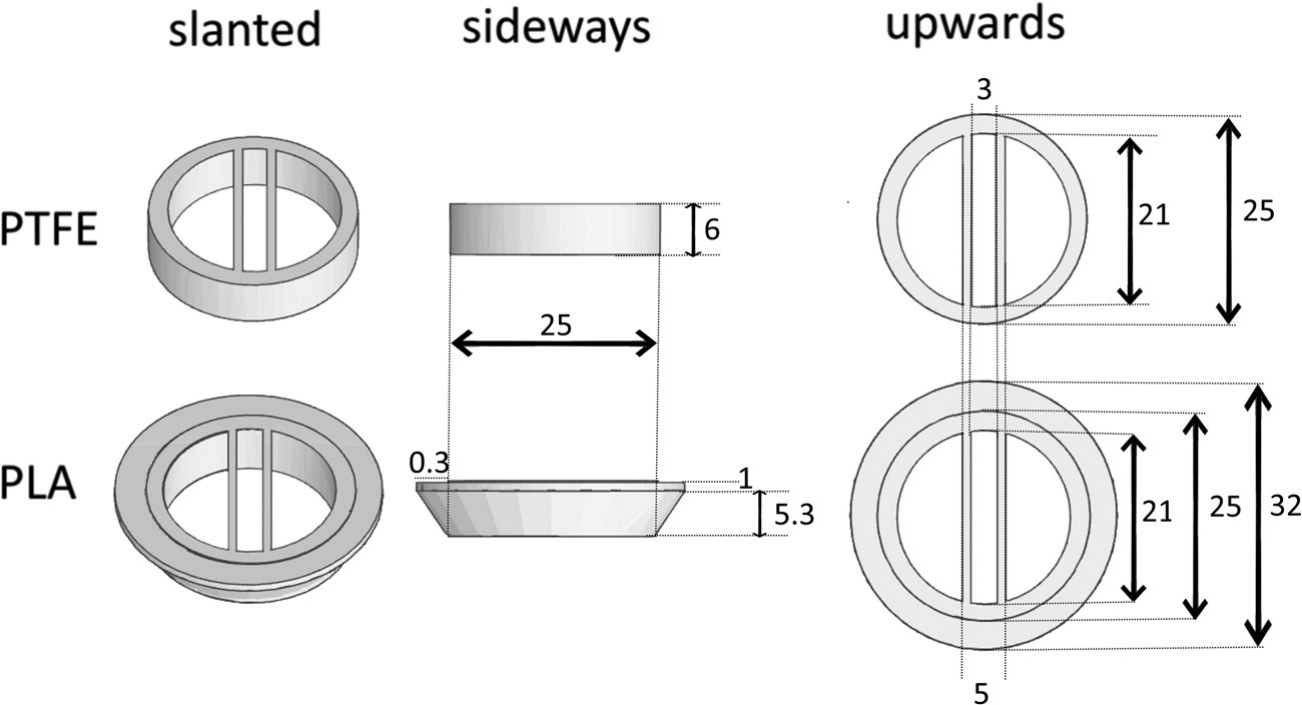
The shapes of the Campenot chamber. To match the mass and shape of the contact surface to the medium, the shape of PLA was modeled as shown. The mass of each chamber is 2.7 g. All size units in the figure are in millimeters.

The circular compartments of the PTFE and PLA Campenot chambers were greased (High Vacuum Grease, Dow Corning Corporation, Michigan, USA) and placed so that the straight partition of the Campenot chambers crossed the straight lines drawn by the pin rake. Three wells were made of conventional PTFE (Teflon), and the remaining were PLA formed by the FDM 3D printer. The shape of the PLA chambers was modeled, as shown in Figure 3, to match the mass and shape of the contact surface to the medium. All the Campenot chambers, including the PTFE ones, weighed 2.7 g.

When cultured, axons pass under the left and right partitions from this central compartment to reach the left and right compartments, resulting in a high concentration of nerve growth factor (NGF).

In this study, for better adhesion of the chambers and reducing the contamination of cell bodies to the side compartments, 75 g of aluminum rods were placed as weights in each chamber for 2 h before the cells were added.

### 2.3 Animals

Male C57BL/6 mice aged 6–16 weeks were used in this study. The animals were maintained under a 12-h light/dark cycle (daytime from 7 am to 7 pm) at a constant temperature. All the mice were euthanized with sevoflurane. Six chambers were allocated to one mouse, and three mice were used for each PLA group. Therefore, each group had nine PTFE chambers and nine PLA chambers.


**Ethical approval:** The research related to animal use has been complied with all the relevant national regulations and institutional policies for the care and use of animals and has been approved by the Animal Experimentation Ethics Committee of the Kyoto Prefectural University of Medicine.

### 2.4 Medium

Dulbecco’s phosphate-buffered saline (DPBS) was supplemented with 1% penicillin–streptomycin. From now on, this will be referred to as “modified DPBS.”

Dulbecco’s modified Eagle’s medium (DMEM) supplemented with 10% bovine serum and 1% penicillin–streptomycin was used during cell isolation. From now on, this will be referred to as “modified DMEM.”

Neurobasal (NB) plus medium supplemented with 2% B27 supplement, 1% penicillin–streptomycin, and 0.5% lactose were used for the culture. From now on, this will be referred to as “modified NB plus medium.”

NGF, glial cell line-derived neurotrophic factor (GDNF), and arabinocytidine (Ara-C) were added to modified NB plus medium depending on the situation.

NB plus medium and DMEM contain phenol red.

### 2.5 Preparation of primary neuronal culture

Principal neurons were derived from the dorsal root ganglia (DRGs), which were dissociated by a mechanical procedure from C57BL/6 J mice.

**Figure 4:**
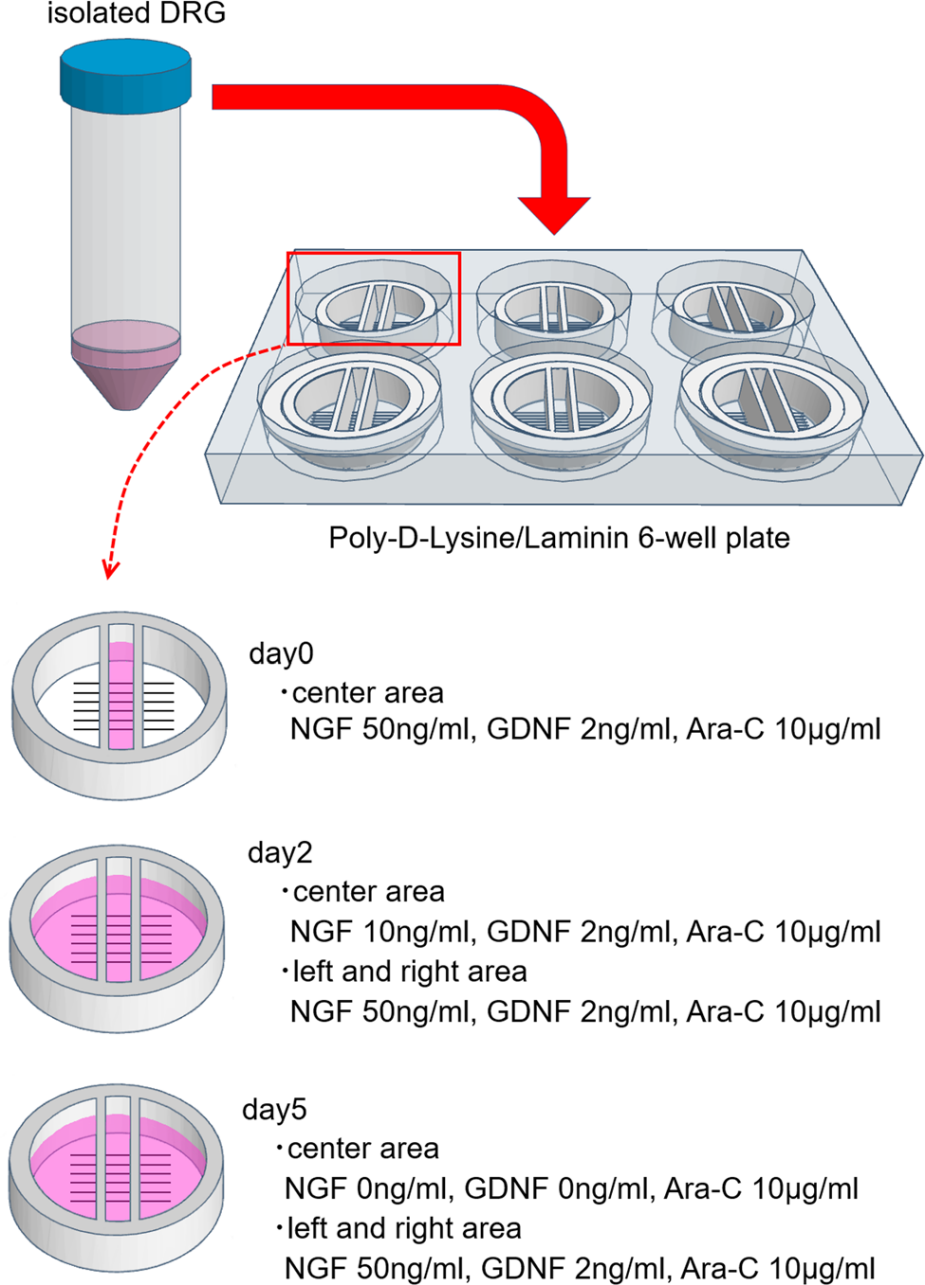
Changes in additive concentration. Until day 2, both side compartments are kept empty to prevent neurons from flowing there. To promote axon extension, the concentration of NGF in the center chamber was reduced by every medium change. GDNF in the center chamber was removed on day 5. The concentrations of the additives in both side compartments were kept at NGF 50 ng/mL and GDNF 2 ng/mL. Until day 2, both side compartments were kept empty to prevent neurons from flowing there.

After euthanizing the mice, fresh DRGs were dissected and quickly transferred into ice-cold modified DPBS in a 10 mL falcon tube. After 3 min of centrifugation at 500×*g* and removing the supernatant, 3 mL of collagenase and dispase solution (collagenase 1 mg/mL, dispase 2.4 U/mL, dissolved in DPBS) was added and incubated at 37°C for 70 min. After 3 min of centrifugation at 500×*g* and washing with 5 mL modified DMEM, 1.9 mL of modified DMEM supplemented with 0.1 mL DNase (dissolved in DPBS) was added to the precipitate. The cells were isolated by pipetting 70 times, using a cell strainer, cell suspension, and 4 mL modified DMEM overlaid on 6 mL of 10% bovine albumin. After 10 min of centrifugation at 1,000×*g* and washing with 5 mL modified DMEM once, the precipitate was suspended in 900 μL modified NB plus medium (NGF 50 ng/mL, GDNF 2 ng/mL, Ara-C 10 μg/mL); 150 μL of the suspension was added to the center compartments of the six Campenot chambers, as shown in Figure 4.

### 2.6 Medium changes in Campenot chamber

On day 2, the medium of the center chamber was changed to modified NB plus medium (NGF 10 ng/mL, GDNF 2 ng/mL, and Ara-C 10 μg/mL); 250 μL of modified NB plus medium (NGF 50 ng/mL, GDNF 2 ng/mL, and Ara-C 10 μg/mL) was added to the compartments on both sides. On day 5, the medium of the center chamber was changed to modified NB plus medium (non-NGF, non-GDNF, and Ara-C 10 μg/mL), and the media on both sides were changed to the same modified NB plus medium (NGF 50 ng/mL, GDNF 2 ng/mL, Ara-C 10 μg/mL). On day 7, each dish was immunostained and observed (Figure 4).

### 2.7 Immunostaining

Nerve cells were immunostained for 7 days after the initiation of the cell culture. The following procedures were performed for each compartment in each chamber:

First, the medium was aspirated and washed with 500 μL of DPBS, and 4% paraformaldehyde was added. After 5 min, the cells were washed with 500 μL of DPBS, and 0.1% Triton X-100 was added. After another 5 min, they were washed with 500 μL of DPBS, and Blocking One (Nacalai Tesque Inc.) was added. After incubation at 37°C for 30 min and aspirating Blocking One, an anti-beta tubulin antibody (cat. no. ab179513; Abcam) was added as the primary antibody (1:1,000). After overnight incubation at 4°C or incubation at 37°C for 1 h, the cells were washed with 500 μL of DPBS, and antibodies with green fluorescent antibody (GFP) (cat. no. ab150077; Abcam) (1:1,000) were added as secondary antibodies. 4′,6-Diamidino-2-phenylindole (DAPI) (1:20) was also added to the secondary antibody solution. After overnight incubation at 4°C or incubation at 37°C for 1 h, the cells were washed with 500 μL of DPBS and observed.

**Figure 5:**
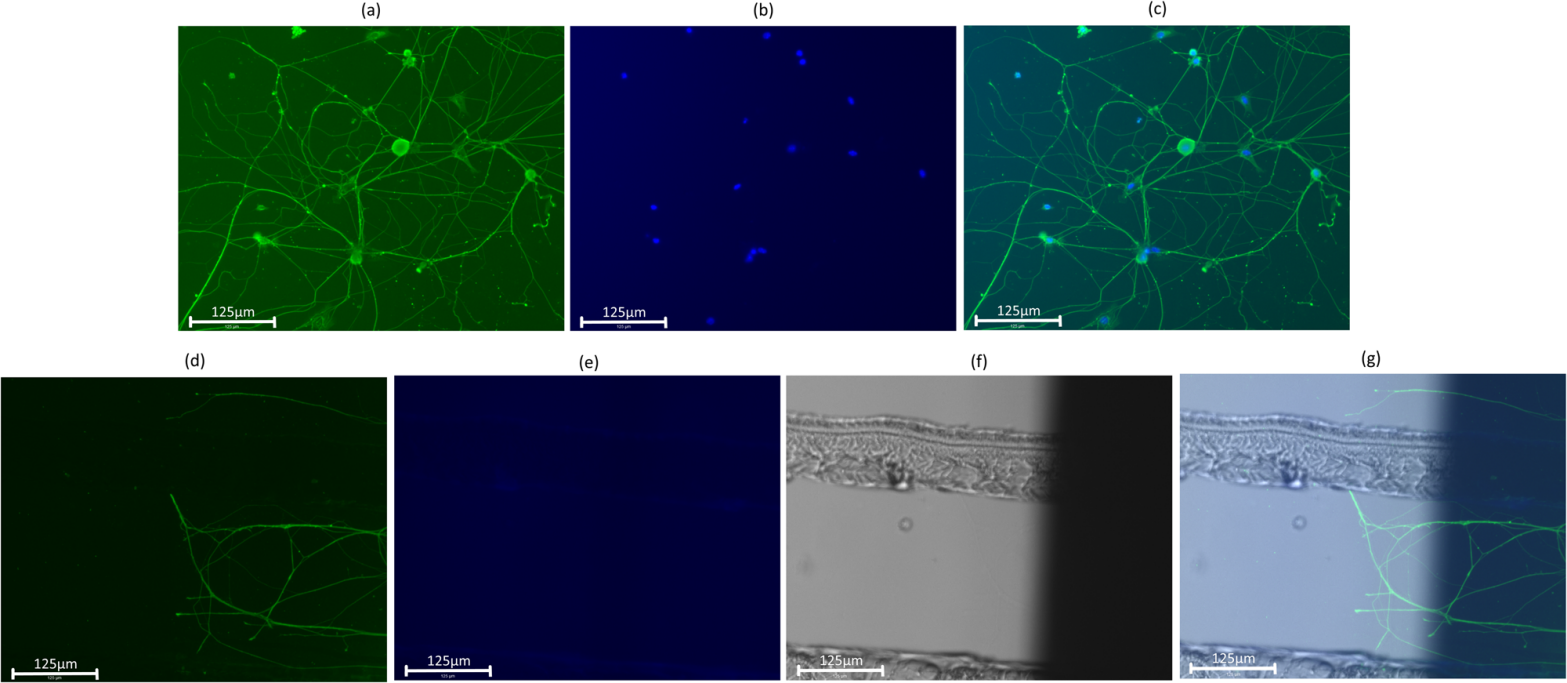
Merged image. The size of the scale bar is 125 μm. Axons are immunostained with anti-beta tubulin with GFP and observed at a wavelength of 482 nm (a). Cell bodies are immunostained with DAPI and observed at a wavelength of 390 nm (b). These images are merged for observation (c). When axon extension was observed, the three images for GFP, DAPI, and the ordinal microscope view were merged (d–g).

**Figure 6:**
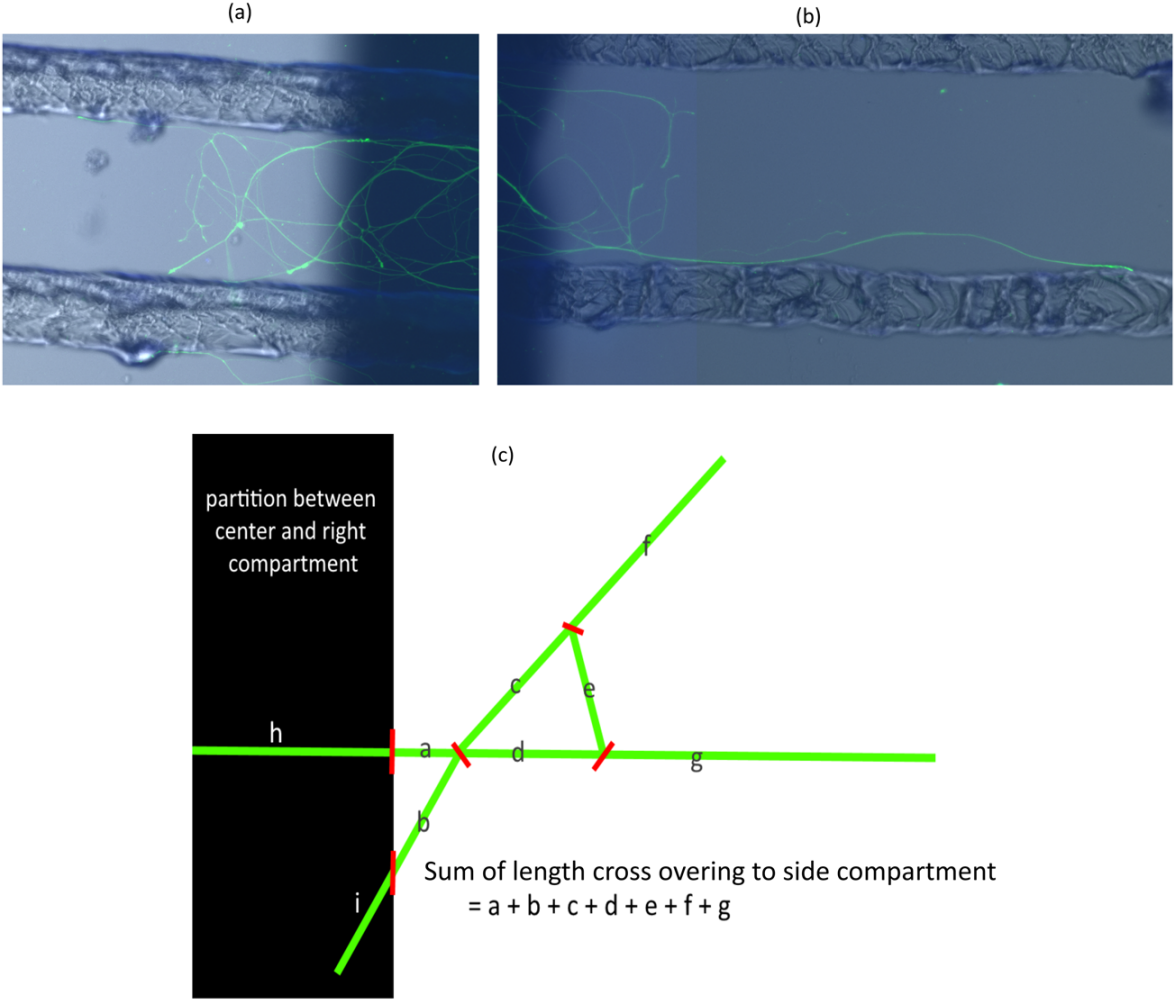
Measurement of axon extensions to both side compartments. The shape of the axon differs from axon to axon. Some axons are mesh-like (a) and others are long and straight (b). To evaluate the axon extension, the sum of the crossed-over axon lengths measured using ImageJ was used (c).

Beta-tubulin was observed at a wavelength of 482 nm, and the cell body was observed at a wavelength of 390 nm using the EVOS FLoid Imaging System. These two images were merged to confirm axons and cell bodies in the central compartment (Figures 5a, 6b and c). When observing the extension of axons to the left and right compartments, an ordinal microscope view was added to check the absence of the cell body (Figures 5d and 6e–g).

### 2.8 Observation

The axons extending to the left and right compartments may have a mesh-like structure, as shown in Figure 6a, or extended, as shown in Figure 6b. As shown in Figure 6c, the degrees of extension to the left and right compartments were evaluated by adding all the lengths of the axons that did not have cell bodies in the left and right compartments. For each chamber, the sum of the axon lengths crossing to both side compartments was the subject of statistical analysis. The length was measured using ImageJ software.

### 2.9 Measuring pH and leakage

Each greased chamber (PTFE, white PLA, natural PLA) was placed on a scratched 35 mm Petri dish and a 75 g aluminum rod was placed on it for 2 h to make it stick. Eight chambers were prepared for each group. For each group of four chambers, 150 µL of modified NB plus medium (NGF 50 ng/mL, GDNF 2 ng/mL, and Ara-C 10 μg/mL) was added to the central compartment and 250 µL to the left and right compartments (pH group), and for the remaining 4,150 µL of pure water was added to the central compartment and 250 µL of modified NB plus medium (NGF 50 ng/mL, GDNF 2 ng/mL, and Ara-C 10 μg/mL) was added to the left and right compartments (leakage group). As a control, 1000 µL of modified NB plus medium (NGF 50 ng/mL, GDNF 2 ng/mL, and Ara-C 10 μg/mL) was placed on a 35 mm Petri dish (control group). Four dishes were prepared for the control group. All Petri dishes were incubated at 37°C.

After 72 h, the pH of the central compartment of each chamber was measured for the pH group and control group. For the leakage group, the absorbance of each compartment was measured using an absorptiometer and the absorbance at a wavelength of 560 nm was recorded [27]. Based on the measured values, the amount of leakage was estimated by dividing the average of the absorbance of the left and right compartments by the absorbance of the central compartment.

### 2.10 Statistical analysis

The extensions of the axons of the PTFE group and PLA groups were compared using the Wilcoxon signed-rank sum test. The Kruskal–Vallis test was used to compare pH and absorbance, and the Mann–Whitney *U* test with Holm’s multiple comparisons, was used to compare the differences between the two groups. Two-sided tests were performed with a significance level of 95%. All the statistical analyses were performed using EZR (Saitama Medical Center, Jichi Medical University) ver1.40, which is a graphical user interface for R.

## 3 Result

In this study, Campenot chambers were used to confirm the effect of chambers made of PTFE and PLA using an FDM 3D printer based on the degree of nerve cell extension. Two types of PLA, white PLA made by Creality 3D and natural PLA made by Polymaker, were used.

### 3.1 Comparison of axon extension

**Figure 7:**
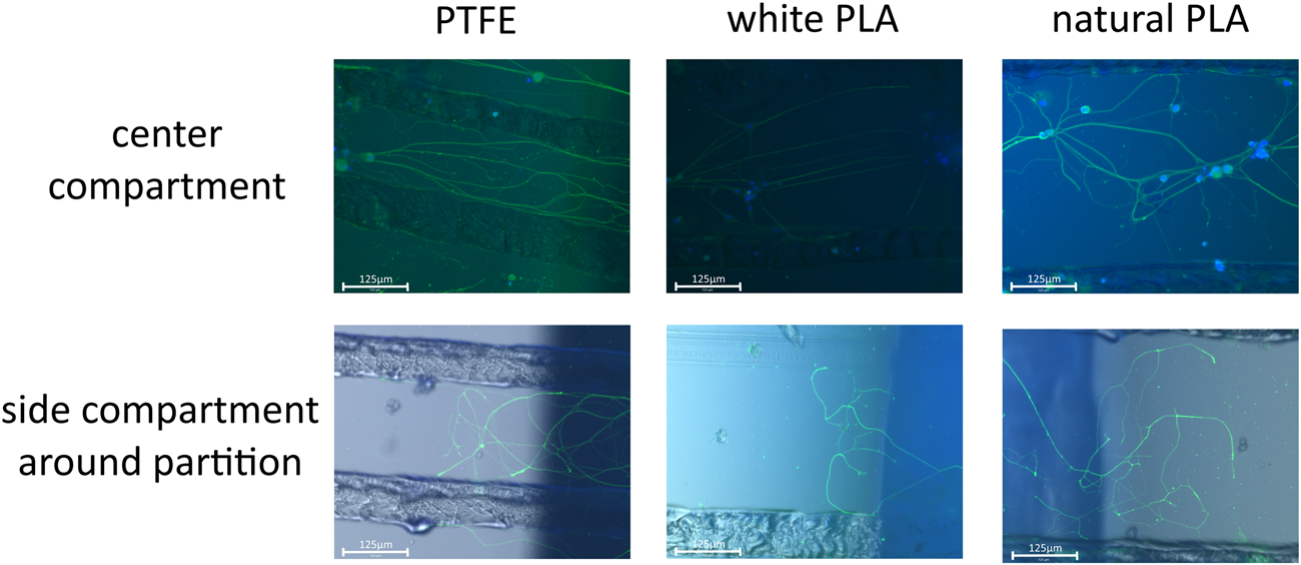
Cultured neurons for each group. The pictures are for cultured neurons and the size of the scale bar is 125 μm. On day 7, beta-tubulin was immunostained to observe the axon, and the cell body was stained by DAPI. Beta-tubulin was observed with GFP. The upper row is lined with images of the center compartment, which are merged with GFP and DAPI. The lower row is lined with images of the side compartment around the partition between the center and side compartments, which are merged with GFP, DAPI, and ordinal microscope view. The columns represent PTFE, white PLA, and natural PLA in that order.

Axon extensions to the left and right compartments were observed for the PTFE, white PLA, and natural PLA groups to a greater or lesser extent (Figure 7).

**Figure 8:**
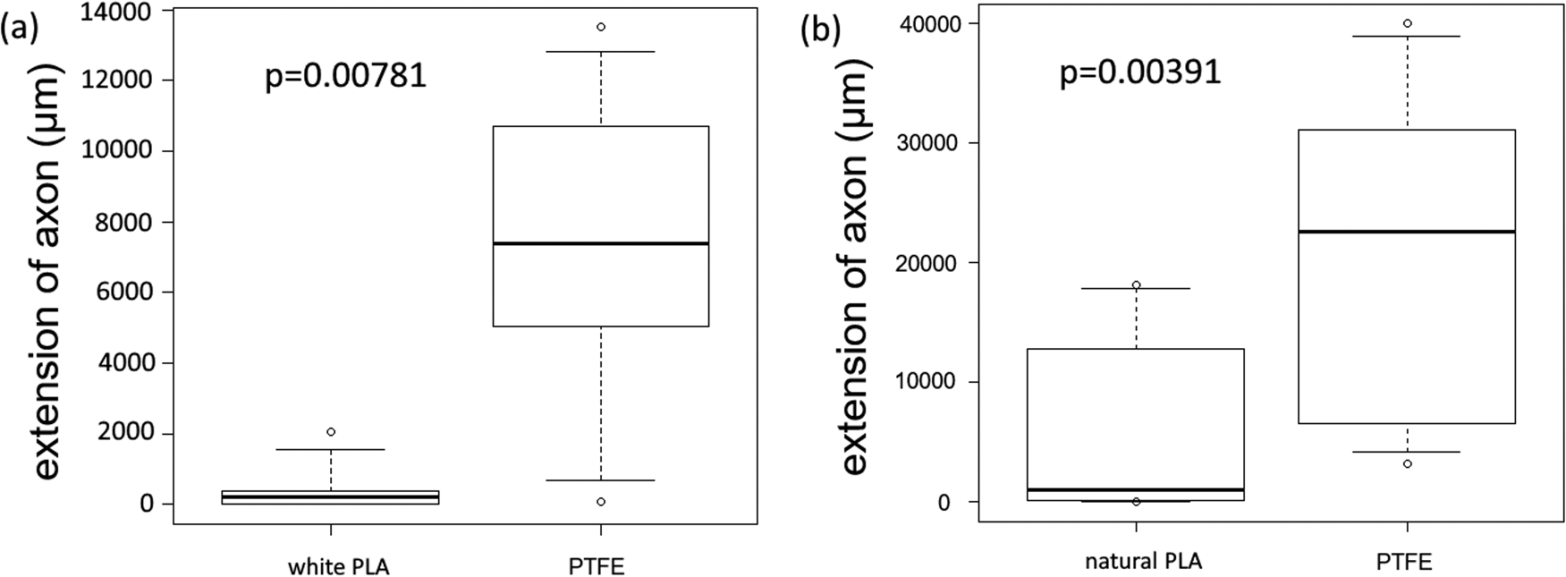
Comparison of PTFE and PLA. The PTFE and PLA groups were analyzed using the Wilcoxon signed-rank sum test, but the box plots were provided for reference. Both comparisons showed significant axon extensions for the PTFE group (white PLA *p* = 0.00781, natural PLA *p* = 0.00391).

A significant extension was observed in the PTFE group compared to the white PLA group (*n* = 9 in each group, *p* = 0.00781). The rank-sum test was used this time; however, the average value of axon extension per chamber was 7,306 μm for the PTFE group and 408 μm for the PLA group (Figure 8a), and four of the nine PLA chambers showed no extensions of axons to the left or right compartments.

The results of the Wilcoxon signed-rank-sum test were the same for the PTFE and natural PLA groups (*n* = 9 in each group, *p* = 0.00391), and the average values of axon extension per chamber were 21,184 and 5,733 μm, respectively (Figure 8b). One of the nine PLA chambers showed no extension of the axons to the left or right compartments.

For both groups, axon extensions to the left and right compartments were observed in all PTFE chambers, although there was variability.

### 3.2 Comparison of pH

**Figure 9:**
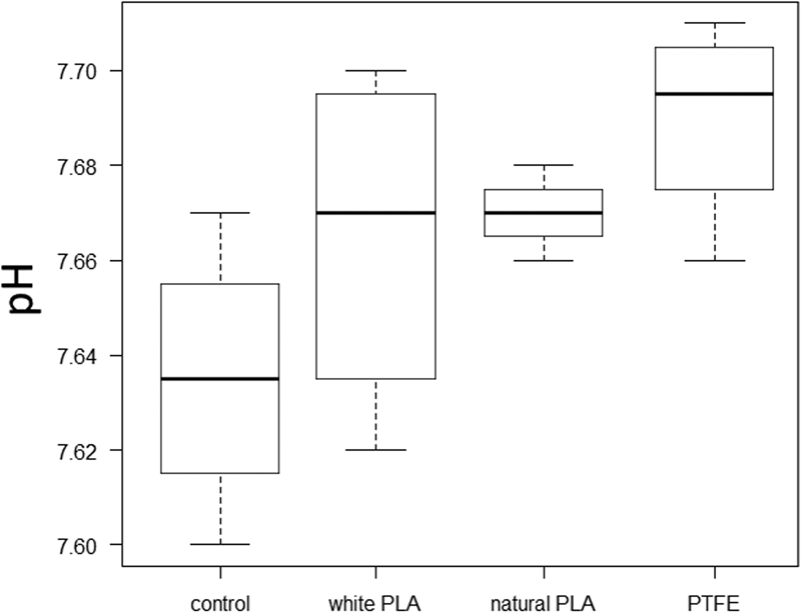
pH of each group. There was no significant difference in the comparison of each group.

Modified NB plus medium was added to each type of chamber and the pH values were compared. The pH in each group was compared as shown in Figure 9. The comparison of the four groups including the control group was *p* = 0.115, and the comparison of the three groups excluding the control group was *p* = 0.347, showed no significant difference.

### 3.3 Comparison of leakage

Pure water was added to the central compartment and modified NB plus medium containing phenol red was added to the side compartment, and the absorbance was measured. The amount of leakage was estimated by dividing the absorbance of the central compartment from the average of the side absorbances.

**Figure 10:**
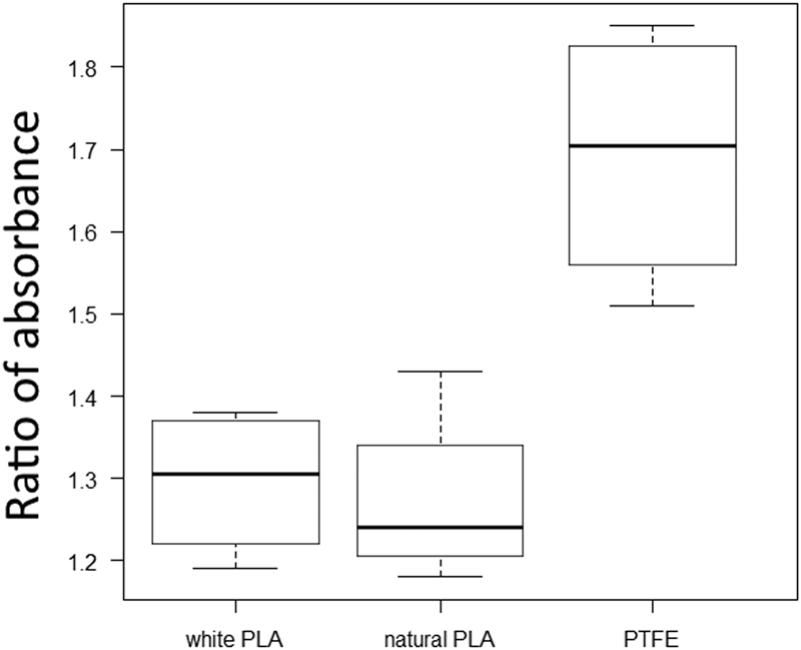
Ratio of absorbance of each group. There was a significant difference between the three groups (*p* = 0.0231), but not between each group. The larger the ratio, the larger the concentration difference, that is, less leakage.

The results are shown in Figure 10. There was a large difference in the mean values of each group (PTFE: 1.69, white PLA: 1.30, natural PLA: 1.27). The Kruskal–Vallis test showed a significant difference between the three groups (*p* = 0.0231), but the Mann–Whitney *U* test with Holm’s multiple comparisons showed a slightly higher *p*-value (PTFE vs white PLA: *p* = 0.086, PTFE vs natural PLA: *p* = 0.086, white PLA vs natural PLA: *p* = 0.772).

## 4 Discussion

In general, white is the basic color of PLA filaments and the color of PLA is transparent; therefore, natural-colored filaments have higher purity and low crystallinity when they are formed by an FDM 3D printer [26]. We attempted to use both types of PLA. Because the extension of axons differs from cell to cell, the Wilcoxon signed-rank sum test was used to compare the results of the neurons obtained from the same mouse.

The conventional PTFE chambers showed greater axon elongation than the PLA chambers created by the FDM 3D printer. The longer elongation is expected to be due to an increase in the number of axons that elongate after responding well to NGF [28].

Recently, culturing with PLA nanosheets has often been conducted [29], and the high hydrophobic property of PLA requires pretreatment for better cell adhesion [30–32]; however, PLA is thought to be biosafe for neurons. In this study, PLA was not pretreated because it was not a scaffold, and the hydrophobic property of PLA may have affected cell extension even though it was not used as a scaffold.

PLA is known for its accelerated degradation by hydrothermal reactions and its degradation by prolonged UV exposure, resulting in organic acids. Therefore, there is a possibility that the sterilization process in this experiment may have been harmful to the cells. PLA is degraded to produce the monomer lactic acid, which contains several organic acids and other compounds [33]. In order to confirm the effect of this, we investigated the pH. However, the difference was not obvious, and no effect of sterilization on neuronal growth was suggested.

3D printers create structures by laminating molten filaments, and there may be slight irregularities on the surface of the PLA chambers created by the 3D printer. PLA and PTFE have different physical properties, such as friction coefficient and surface roughness [34–37]. The coefficient of friction of PTFE is very small [38] and its surface is smooth [39]. On the other hand, the surface of PLA formed by the 3D printer has a rough structure at the microscopic level [40]. PLA is known to have slight distortions due to shrinkage during the formation process. In this study, more leakage was observed in the PLA group compared to the PTFE group. This result suggests that the rough surface or shrinkage of the PLA formed by the 3D printer has a weaker ability to partition the solution compared to PTFE, which may have prevented the concentration difference in the Campenot chamber from working effectively. If that is the cause, then frequent medium changes may give better results. However, the toxicity of PLA itself cannot be denied from this study. Further research is needed.

## 5 Conclusions

When comparing PTFE and 3D-printed PLA Campenot chambers, it was found that better neurite outgrowth was obtained with PTFE. The reason for this may be due to the smoothness of the PTFE surface.

However, this study was only validated with a specific instrument shape, and only limited conclusions can be drawn. More research is needed.

## References

[1] Yoshimura T. Present status and prospects of the 3D printer making educational materials of a molecular model that we can touch. J Technol Educ. 2014;21:53–62.

[2] Tan Z, Liu T, Zhong J, Yang Y, Tan W. Control of cell growth on 3D-printed cell culture platforms for tissue engineering. J Biomed Mater Res A. 2017;105(12):3281–92. 10.1002/jbm.a.36188 28865175

[3] Byrley P, George BJ, Boyes WK, Rogers K. Particle emissions from fused deposition modeling 3D printers: Evaluation and meta-analysis. Sci Total Env. 2019;655:395–407. 10.1016/j.scitotenv.2018.11.070 PMC8350970 30471608

[4] Tedla G, Jarabek AM, Byrley P, Boyes W, Rogers K. Human exposure to metals in consumer-focused fused filament fabrication (FFF)/3D printing processes. Sci Total Env. 2022;814:152622. 10.1016/j.scitotenv.2021.152622 PMC8961686 34963600

[5] Kreß S, Schaller-Ammann R, Feiel J, Priedl J, Kasper C, Egger D. 3D printing of cell culture devices: Assessment and prevention of the cytotoxicity of photopolymers for stereolithography. Mater (Basel). 2020;13(13):3011. 10.3390/ma13133011 PMC7372443 32640644

[6] Oskui SM, Diamante G, Liao C, Shi W, Gan J, Schlenk D, et al. Assessing and reducing the toxicity of 3D-printed parts. Env Sci Technol Lett. 2016;3(1):1–6.

[7] Macdonald NP, Zhu F, Hall CJ, Reboud J, Crosier PS, Patton E, et al. Assessment of biocompatibility of 3D printed photopolymers using zebrafish embryo toxicity assays supplementary information. Lab Chip. 2016;16(2):291–7. 10.1039/c5lc01374g PMC4758231 26646354

[8] Farcas MT, Stefaniak AB, Knepp AK, Bowers L, Mandler WK, Kashon M, et al. Acrylonitrile butadiene styrene (ABS) and polycarbonate (PC) filaments three-dimensional (3-D) printer emissions-induced cell toxicity. Toxicol Lett. 2019;317:1–12. 10.1016/j.toxlet.2019.09.013 PMC7490755 31562913

[9] Wardyn JD, Sanderson C, Swan LE, Stagi M. Low cost production of 3D-printed devices and electrostimulation chambers for the culture of primary neurons. J Neurosci Methods. 2015;251:17–23. 10.1016/j.jneumeth.2015.05.001 PMC4509708 25962333

[10] Naftalovich R, Naftalovich D, Greenway FL. Polytetrafluoroethylene ingestion as a way to increase food volume and hence satiety without increasing calorie content. J Diabetes Sci Technol. 2016;10:971–6. 10.1177/1932296815626726 PMC4928218 26810925

[11] Gritsch L, Conoscenti G, La Carrubba V, Nooeaid P, Boccaccini AR. Polylactide-based materials science strategies to improve tissue-material interface without the use of growth factors or other biological molecules. Mater Sci Eng C Mater Biol Appl. 2019;94:1083–101. 10.1016/j.msec.2018.09.038 30423690

[12] Abdel-Rahman MA, Tashiro Y, Sonomoto K. Lactic acid production from lignocellulose-derived sugars using lactic acid bacteria: overview and limits. J Biotechnol. 2011;156:286–301. 10.1016/j.jbiotec.2011.06.017 21729724

[13] Kimura T, Ishida Y, Ihara N, Saito Y. Degradability of biodegradable plastic poly lactic acid products. JSAM. 2020;64:115–20.

[14] Adsul MG, Varma AJ, Gokhale DV. Lactic acid production from waste sugarcane bagasse derived cellulose. Green Chem. 2007;9(1):58–62.

[15] Rasal RM, Janorkar AV, Hirt DE. Poly (lactic acid) modifications. Prog Polym Sci. 2010;35(3):338–56.

[16] Lu Y, Dong S, Zhang P, Liu X, Wang X. Preparation of a polylactic acid knitting mesh for pelvic floor repair and in vivo evaluation. J Mech Behav Biomed Mater. 2017;74:204–13. 10.1016/j.jmbbm.2017.05.034 28618328

[17] Igaki K, Iwamoto M, Yamane H, Saito K. Development of novel biodegradable poly (L-lactic acid) stent. J Soc Mater Sci Jpn. 2000;49:1030–5.

[18] Van Alst M, Eenink MJD, Kruft MAB, Van Tuil R. ABC’s of bioabsorption: Application of lactide based polymers in fully resorbable cardiovascular stents. EuroIntervention. 2009;5(Suppl F):F23–7. 10.4244/EIJV5IFA4 22100672

[19] Tyler B, Gullotti D, Mangraviti A, Utsuki T, Brem H. Polylactic acid (PLA) controlled delivery carriers for biomedical applications. Adv Drug Deliv Rev. 2016;107:163–75. 10.1016/j.addr.2016.06.018 27426411

[20] Xiao L, Wang B, Guang Yang MG. Poly (lactic acid)-based biomaterials: synthesis, modification and applications. Biomed Sci Eng Technol. 2012;11:247–82.

[21] Sölmann S, Rattenholl A, Blattner H, Ehrmann G, Gudermann F, Lütkemeyer D, et al. Mammalian cell adhesion on different 3D printed polymers with varying sterilization methods and acidic treatment. AIMS Bioeng. 2020;8(1):25–35.

[22] Nishihara D, Iwamatsu Y, Hirata M, Kindaichi KKM. Cell mobility of human periodontal ligament fibroblasts cultured on bioabsorbable materials. Jpn J Conserv Dent. 2005;48:508–16.

[23] Campenot RB. Local control of neurite development by nerve growth factor. Proc Natl Acad Sci U S A. 1977;74(10):4516–9. 10.1073/pnas.74.10.4516 PMC431975 270699

[24] Jadhav DA, Wei L, Shi P. Compartmentalized platforms for neuro-pharmacological research. Curr Neuropharmacol. 2015;14(1):72–86. 10.2174/1570159X13666150516000957 PMC4787287 26813122

[25] Bertrand J, Winton MJ, Rodriguez-Hernandez N, Campenot RB, McKerracher L. Application of Rho antagonist to neuronal cell bodies promotes neurite growth in compartmented cultures and regeneration of retinal ganglion cell axons in the optic nerve of adult rats. J Neurosci. 2005;25(5):1113–21. 10.1523/JNEUROSCI.3931-04.2005 PMC6725958 15689547

[26] Wittbrodt B, Pearce JM. The effects of PLA color on material properties of 3-D printed components. Addit Manuf. 2015;8:110–6.

[27] Rovati L, Fabbri P, Ferrari L, Pilati F. Plastic optical fiber pH SENSOR Using a sol–gel sensing matrix. Fiber optical sensors. London, UK: InTech Open; 2012. p. 415–38.

[28] Tucker BA, Rahimtula M, Mearow KM. Laminin and growth factor receptor activation stimulates differential growth responses in subpopulations of adult DRG neurons. Eur J Neurosci. 2006;24(3):676–90. 10.1111/j.1460-9568.2006.04963.x 16930399

[29] Otomo A, Ueda MT, Fujie T, Hasebe A, Suematsu Y, Okamura Y, et al. Efficient differentiation and polarization of primary cultured neurons on poly(lactic acid) scaffolds with microgrooved structures. Sci Rep. 2020;10(1):6716. 10.1038/s41598-020-63537-z PMC7174324 32317746

[30] Lin Y, Wang L, Zhang P, Wang X, Chen X, Jing X, et al. Surface modification of poly(L-lactic acid) to improve its cytocompatibility via assembly of polyelectrolytes and gelatin. Acta Biomater. 2006;2(2):155–64. 10.1016/j.actbio.2005.10.002 16701873

[31] Wan Y, Yang J, Yang J, Bei J, Wang S. Cell adhesion on gaseous plasma modified poly-(L-lactide) surface under shear stress field. Biomaterials. 2003;24(21):3757–64. 10.1016/s0142-9612(03)00251-5 12818548

[32] Fujie T, Ricotti L, Desii A, Menciassi A, Dario P, Mattoli V. Evaluation of substrata effect on cell adhesion properties using freestanding poly(L-lactic acid) nanosheets. Langmuir. 2011;27:13173–82. 10.1021/la203140a 21913651

[33] Inkinen S, Hakkarainen M, Ann-Christine Albertsson AS. From lactic acid to poly(lactic acid) (PLA): characterization and analysis of PLA and its precursors. Biomacromolecules. 2011;12(3):523–32. 10.1021/bm101302t 21332178

[34] Yang H, Ji F, Li Z, Tao S. Preparation of hydrophobic surface on PLA and ABS by fused deposition modeling. Polym (Basel). 2020;12(7):1539. 10.3390/polym12071539 PMC7407596 32664645

[35] Busscher HJ, Stokroos I, Van Der Mei HC, Rouxhet PG, Schakenra AdJM. Preparation and characterization of superhydrophobic FEP-Teflon surfaces. J Adhes Sci Technol. 1992;6(3):347–56.

[36] Pawlak W. Wear and coefficient of friction of PLA-graphite composite in 3D printing technology. Eng Mech. 2018;649–52.

[37] Fetfatsidis KA, Gamache LM, Gorczyca JL, Sherwood JA, Jauffrès D, Chen J. Design of an apparatus for measuring tool/fabric and fabric/fabric friction of woven-fabric composites during the thermostamping process. Int J Mater Form. 2013;6:1–11.

[38] Burton JC, Taborek P, Rutledge JE. Temperature dependence of friction under cryogenic conditions in vacuum. Tribol Lett. 2006;23:131–7.

[39] Qian Y, Chi L, Zhou W, Yu Z, Zhang Z, Zhang Z, et al. Fabrication of TiO2 – modified polytetrafluoroethylene ultrafiltration membranes via plasma-enhanced surface graft pretreatment. Appl Surf Sci. 2016;360:749–57.

[40] Kirill SE, Evgeniy GG, Valentine PA. Revealing interactions of layered polymeric materials at solid-liquid interface for building solvent compatibility charts for 3D printing applications. Sci Rep. 2019 Dec 27;9(1):20177. 10.1038/s41598-019-56350-w PMC6934857 31882642

